# Nutrigenetic Tailored Diet Results in Long-Term (12 Months) Weight-Loss Compliance and Improved Cardiometabolic Outcomes in a Real-World Community-Based Setting

**DOI:** 10.3390/nu18162618

**Published:** 2026-08-10

**Authors:** Mor Bensimhon, Alice H. Lichtenstein, Ruth Birk

**Affiliations:** 1 Nutrition Department, Health Science Faculty, Ariel University, Ariel 40700, Israel; morbensim@gmail.com; 2 Jean Mayer USDA Human Nutrition Research Center on Aging, Tufts University, Boston, MA 02111, USA

**Keywords:** nutrigenetics, personalized nutrition, weight loss, metabolic health, obesity, real-world

## Abstract

**Background/Objectives**: Individual responses to weight-loss diets vary widely due to genetic, metabolic, and lifestyle factors, motivating individualized approaches over “one-size-fits-all” advice. We aimed to evaluate long-term (12-month) weight loss, weight maintenance, and cardiometabolic outcomes of a cohort following a nutrigenetically tailored Mediterranean-style dietary program in a real-world, community setting. **Methods**: The retrospective data cohort included 426 Israeli adults (mean BMI 32.11 ± 4.58 kg/m^2^; 66.9% women) who participated in a nutrigenetic weight-loss program. Following nutrigenetic direct-to-consumer screening of 425 genetic variants, the participants received a 3-month personalized adjusted diet with remote dietitian-led follow-up by telephone, after which they transitioned to a 9-month self-monitoring and maintenance period. Blood biochemical parameters were reported from routine Health Maintenance Organization (HMO) laboratory testing. Weight/BMI trajectories (0–3, 3–6, 6–9, 9–12 months) were evaluated using linear mixed-effects models; biomarker changes were analyzed with repeated-measures MANOVA and follow-up univariate ANOVAs. **Results**: Model-estimated cumulative weight loss at 12 months was 10.12 kg (11.4% of baseline) with a mean BMI reduction of 3.68 kg/m^2^ (*p* < 0.001). The steepest decline occurred during months 3–6 (−1.13 kg/month). Triglycerides (−14.06 mg/dL), total cholesterol (−4.68 mg/dL), LDL cholesterol (−2.49 mg/dL), fasting glucose (−5.14 mg/dL), and HbA1c (−0.14%) improved significantly (*p* < 0.05), while HDL cholesterol levels remained stable (+0.86 mg/dL; *p* > 0.05) at 12 months. Among participants with elevated baseline biochemical values, levels transitioned to normal range by 12-month follow-up for triglycerides (46.4%), total cholesterol (29.4%), LDL cholesterol (34.5%), glucose (39.6%), and HbA1c (35.5%). **Conclusions**: In a real-world cohort, inclusion of genetic information to personalize dietary guidance was implemented through a flexible, individually tailored program supported by an initial 3-month phase of remote dietitian-led counseling. This approach was associated with durable, clinically meaningful reductions in weight and cardiometabolic risk markers over 12 months. Randomized trials are warranted to confirm causality and evaluate cost-effectiveness.

## 1. Introduction

Common obesity is a multifactorial, chronic condition arising from the dynamic interplay between genetic and environmental factors. Twin, adoption, and family studies attribute 40–70% of the variance in body weight to genetic background [1], while the remainder reflects modifiable environmental exposures such as energy-dense ultra-processed diets [2], sedentary lifestyles, and psychosocial factors [3,4,5,6]. Globally, approximately 2.5 billion adults are living with overweight, and about 890 million are living with obesity [7,8]. Excess adiposity markedly elevates the risk of cardiovascular disease, type 2 diabetes, musculoskeletal disorders, and several cancers [9,10,11,12].

Common obesity is a polygenic multifactorial trait, where the cumulative influence of hundreds of variants predisposes individuals to obesity risk [13,14]. High-throughput genotyping, genome-wide association studies (GWAS), and clinical and basic research have identified common obesity-predisposing variants, which are involved in many metabolic pathways, including satiety and appetite regulation, adipocyte biology, energy expenditure, and the metabolism of specific macronutrients, particularly lipids and carbohydrates [15,16]. Individuals with high polygenic risk are more susceptible to weight gain and are at a higher risk of developing obesity when exposed to an obesogenic environment characterized by energy-dense foods and sedentary behavior [17,18,19].

Structured lifestyle change is the cornerstone of obesity treatment; however, long-term adherence and maintenance are poor. Conventional hypocaloric regimens, including low-fat, low-carbohydrate, and Mediterranean-style diets, typically result in a 5–10% reduction in baseline weight within 6 months, the standard accepted requirement for successful weight reduction [20]. However, maintenance of reduced weight is a challenge, and weight regain often begins before the first-year follow-up [21,22]. Furthermore, meta-analyses of primary-care programs that combine dietary counseling with physical-activity advice report only an additional mean loss of 2–3 kg at 12 months compared with minimal-contact controls, underscoring the limitations of non-individualized approaches [23,24,25].

Importantly, substantial inter-individual variability exists in response to weight loss interventions, even under standardized dietary protocols. While some individuals achieve clinically significant reductions in body weight, others lose little or even regain weight despite similar adherence [26]. For instance, a large systematic review and meta-analysis of randomized controlled trials found wide heterogeneity in individual responses to identical diets, with some participants achieving substantial weight loss while others gained weight, highlighting the substantial individual variability in outcomes [27]. This variability has been attributed both to genetic predispositions, such as variants in *FTO* (fat mass and obesity-associated) or *PPARG* (peroxisome proliferator-activated receptor gamma) influencing weight loss and regain [28,29,30], and to physiological metabolic adaptations, including greater-than-predicted reductions in resting energy expenditure and hormonal responses that blunt the effects of calorie restriction [31,32,33]. Notably, the magnitude of this metabolic adaptation varies substantially between individuals, suggesting that genetic and adaptive mechanisms are complementary rather than mutually exclusive [34,35]. These findings emphasize that conventional, non-individualized approaches often fail to capture the complexity of obesity treatment and strengthen the rationale for precision strategies that tailor interventions to an individual’s genetic and metabolic profile [13,18,36].

Nutrigenetics offers a promising approach by tailoring dietary advice to an individual’s genetic background [14,37], yet relatively few studies have directly evaluated its role in weight loss, and their findings have been mixed. Early observational data suggested that DNA-based diets might improve adherence and promote greater reductions in weight [38], but randomized controlled trials have been largely inconsistent: one reported no overall benefit despite genotype-specific effects [39], the large DIETFITS trial found no genotype–diet interactions [26], and while the pragmatic NOW trial reported modest benefits and improved adherence [40,41], the PREVENTOMICS [42] and POINTS [43] trials showed little or no advantage over standard care. In contrast, smaller cohorts within specific populations have shown promising results [44,45]. Overall, given the variation in study designs, the evidence base remains limited, heterogeneous, and inconclusive.

Current proposals for defining personalized nutrition highlight the use of individual data to improve dietary adherence and health status [46]. The present study aimed to assess both weight loss and long-term weight maintenance of a personalized nutrition program, delivered through a direct-to-consumer (DTC) platform, in a real-world, community-based cohort. Specifically, we evaluated weight reduction and cardiometabolic outcomes following an individualized, nutrigenetically tailored diet program based on a Mediterranean-style framework, over a 12-month follow-up period. The Mediterranean-style diet that framed this program reflects the standardized nutritional recommendations of the Israeli Ministry of Health [47,48] and is recognized for its beneficial effect on health [49].

## 2. Materials and Methods

### 2.1. Participants

**Figure 1:**
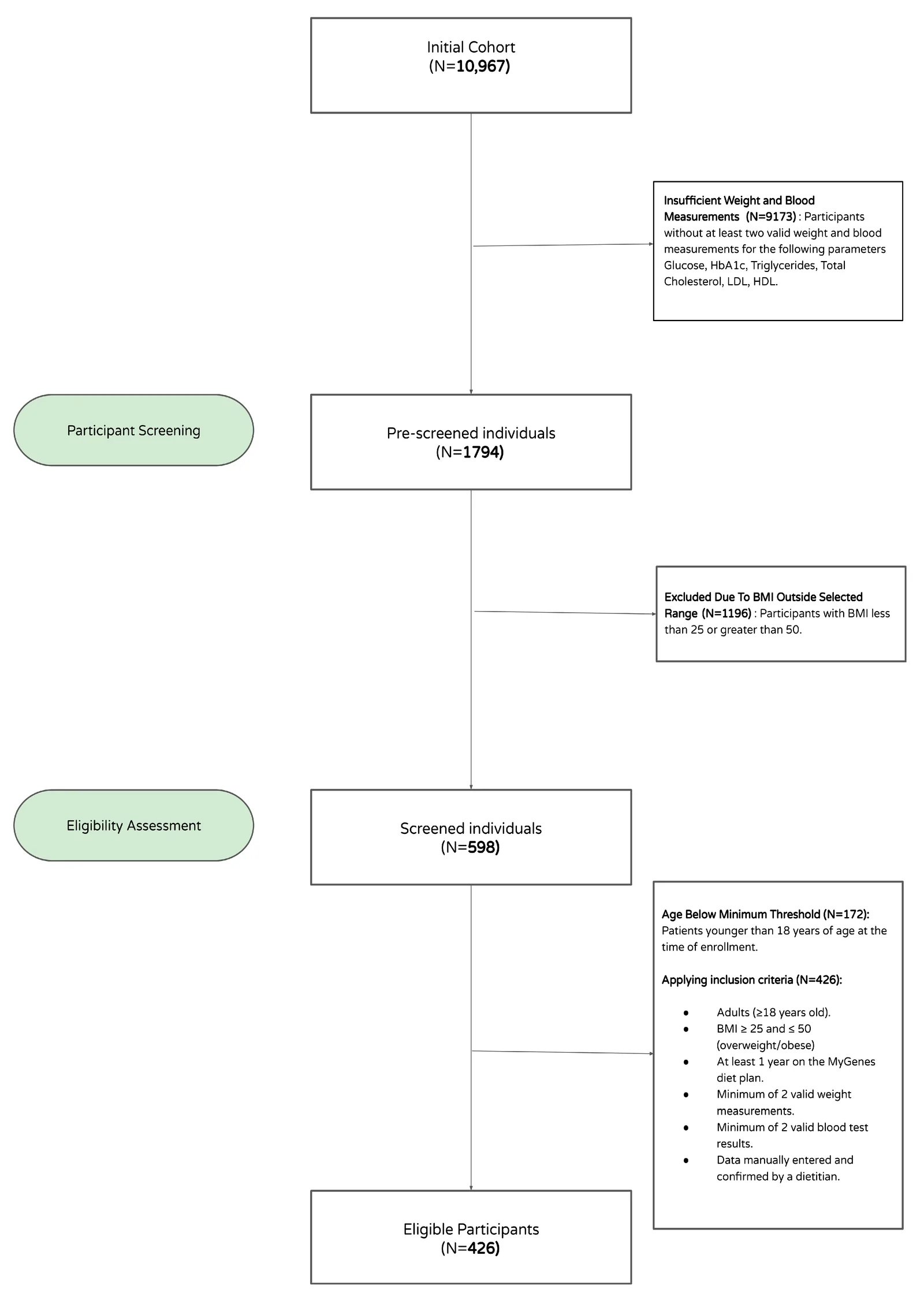
Cohort flowchart.

This retrospective, real-world, community-based data cohort included 426 Israeli adults (66.9% women; age ≥ 18 years) with a mean Body Mass Index (BMI) of 32.11 ± 4.58 kg/m^2^. Participants were adults who had voluntarily purchased the MyGenes (Lev Hai Genetics Ltd., Tel-Aviv, Israel) DTC program, which included the genetic test and the accompanying personalized dietary guidance as a single package; enrollment was self-selected rather than through study recruitment. Data were retrospectively retrieved between December 2020 and June 2024 from the registered Israeli database (#700068969). All data were de-identified and fully anonymized prior to extraction and analysis. Anthropometric and biochemical data were initially collected via a structured, self-administered online questionnaire and the MyGenes platform, and were subsequently validated by clinical dietitians during scheduled interviews. The study was conducted in accordance with the Declaration of Helsinki and the protocol was approved by the Institutional Ethics Committee of Ariel University (Protocol code: AU-HEA-RB-20220214). Inclusion criteria included: adults (≥18 years) with a baseline BMI ranging from 25 to 50 kg/m^2^ (classified as overweight or obesity) who participated in the MyGenes nutrigenetic diet plan for ≥ 12 months, availability of at least two valid weight measurements and two valid blood test records (at baseline and at 12-month follow-up), which were verified by a dietitian. Exclusion criteria were: <18 years, pregnancy, missing anthropometric measurements, or diagnosed with specific genetic or metabolic disorders known to affect weight regulation. Summary of participant selection process is detailed in the flow diagram (Figure 1).

### 2.2. Genetic Testing and Nutrigenetic Profile

Upon enrollment, each participant underwent genetic testing through the MyGenes DTC platform. Genomic DNA was extracted from a saliva sample and genotyped for a panel of 425 single-nucleotide polymorphisms (SNPs) spanning several domains, including: body-weight regulation, lipid metabolism, carbohydrate metabolism, appetite and food-intake regulation, vitamins and micronutrients, food sensitivities, and taste preferences. For each domain, the panel captures common variants that have been associated with the corresponding trait, so that a participant’s combined genotype profile characterizes their predisposition across these domains. Genotyping was performed by the MyGenes service (Lev Hai Genetics Ltd., Tel-Aviv, Israel), drawing on the same Israeli registry (#700068969) and population previously studied by our group [50,51]. For each participant, the genotype profile was translated by the MyGenes algorithm into individually tailored recommendations: for example, variants indicating reduced carbohydrate tolerance, lactose intolerance, or higher obesity risk were used to adjust the corresponding components of the Mediterranean-style base diet.

### 2.3. Nutritional Treatment and Monitoring

Following the genetic test, each participant received a personalized dietary plan. This program was designed to complement standardized nutritional guidance based on the Israeli Ministry of Health National Nutrition Recommendations (specifically the Mediterranean-style diet) [47,48] and guidelines from the World Health Organization (WHO) [52]. The dietary framework (Table S1), including the Mediterranean-style food pyramid [53] (Figure S1), is presented in the Supplementary Materials. The nutritional plan was individually tailored based on the anthropometric, phenotype and nutrigenetic test recommendations and included a 3-month active follow-up period supervised by a registered clinical dietitian. The Mediterranean-style diet served as the common dietary base for all participants, individually modified according to each participant’s nutrigenetic profile (Section 2.2). At the initial nutritional assessment, a registered clinical dietitian reviewed the participants’ reasons for enrolling, personal goals, and habitual behaviors, together with a qualitative dietary anamnesis of habitual food intake. Height and weight were self-entered by the participant and verified together with the dietitian, from which BMI was derived. The dietitian additionally reviewed the participant’s blood biochemical results and individual genetic report and, together with the participant, formulated a personalized action plan aligned with their goals. As part of the initial assessment, participants were encouraged to undergo routine blood biochemical testing through their Health Maintenance Organization (HMO) to evaluate key metabolic biomarkers including a lipid panel comprising triglycerides (TG), total cholesterol, LDL cholesterol, and HDL cholesterol, as well as glycemic markers including fasting glucose and HbA1c, which were reviewed in conjunction with the personalized nutrition plan. Subsequent follow-up sessions during the active phase were conducted at approximately biweekly intervals (approximately six calls over the 3-month active phase) by telephone, focusing on dietary adherence and real-time adjustment of the nutrition plan based on each participant’s preferences, tolerances, and progress, while remaining aligned with their nutrigenetic recommendations. Self-reported weights were also reviewed and verified during these calls. Beyond this active phase, participants continued to self-monitor and enter their weight into the platform during the subsequent maintenance period, which proceeded without active dietitian supervision, and uploaded subsequent routine blood biochemical results to the platform, enabling monitoring over the 12-month period. An overview of the study design and the schedule of assessments is provided in Figure S2 (Supplementary Materials).

### 2.4. Statistical Analysis

All statistical analyses were performed using IBM SPSS Statistics for Windows, Version 28.0 (IBM Corp., Armonk, NY, USA). Longitudinal changes in weight and BMI were evaluated using linear mixed-effects models with random intercepts to account for repeated measures nested within individual participants. Such models are well suited to longitudinal data from real-world cohorts, as they allow baseline values to vary across individuals and use all available observations even when follow-up is unbalanced or incomplete. This within-subject approach was particularly suitable for the present cohort, in which each participant received an individually tailored dietary plan based on their genetic profile; modeling each participant’s change relative to their own baseline allowed outcomes to be evaluated despite the inherent heterogeneity of the personalized interventions. Time was treated as a continuous variable within each interval, and separate models were constructed for each of the four time segments: 0–3 months, 3–6 months, 6–9 months, and 9–12 months. This approach was chosen to accommodate nonlinear patterns of weight loss and to capture differences in weight reduction dynamics across the study timeline. Each model included fixed effects for time, measured in months since the beginning of the respective interval, age at baseline, and sex. In addition, interaction terms between time and age and between time and sex were tested to determine whether the trajectory of weight change differed by age or sex. Model estimation was conducted using the restricted maximum likelihood (REML) approach. Estimated marginal means and corresponding 95% confidence intervals were extracted for interpretation of model results. A *p*-value < 0.05 was considered statistically significant. To assess changes in blood biomarkers (TG, total cholesterol, LDL cholesterol, HDL cholesterol, glucose, and HbA1c), a two-way repeated measures MANOVA was first conducted, with Time serving as the within-subject factor and Sex as the between-subjects factor, to evaluate overall differences across all biomarkers simultaneously. Follow-up univariate mixed repeated measures ANOVAs were then performed for each biomarker individually, with Bonferroni adjustment applied to correct for multiple comparisons. Effect sizes were reported using partial eta squared (partial η^2^). A *p*-value < 0.05 was considered statistically significant.

## 3. Results

### 3.1. Participant Characteristics

The final study cohort included 426 Israeli adults. The mean baseline Body Mass Index (BMI) was 32.11 ± 4.58 kg/m^2^, indicating that the majority of participants were classified as obese at study entry. The cohort was predominantly female (66.9%), with a mean age of 63.21 ± 11.08 years and a mean weight of 88.98 ± 15.90 kg. Baseline demographic, anthropometric, and biochemical characteristics are summarized in Table 1.

### 3.2. Weight Reduction and Maintenance Following Precision Nutrition

**Figure 2:**
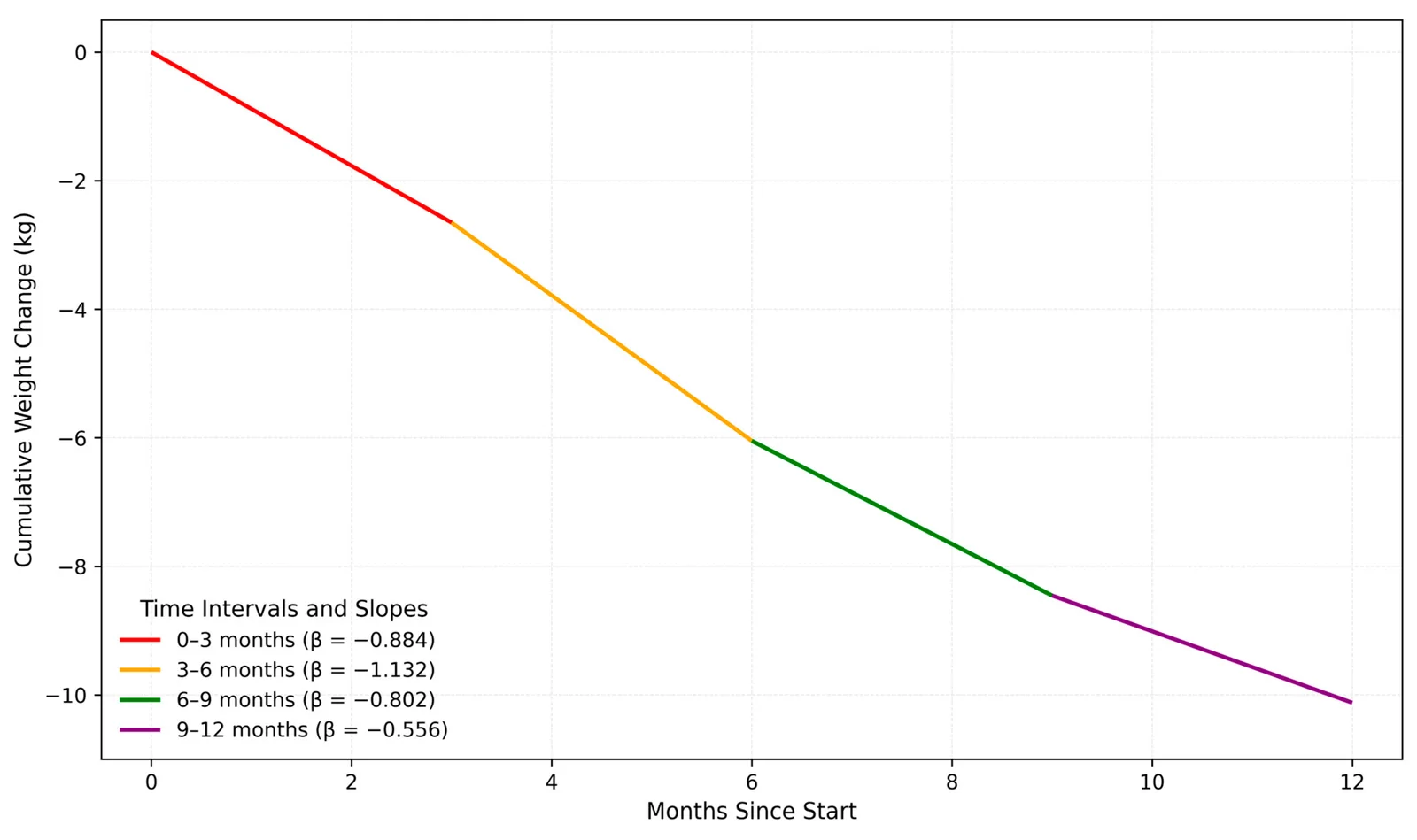
Cumulative weight loss based on linear mixed-effects models. The curves illustrate the mean rates of weight change across the four time intervals (0–3, 3–6, 6–9, and 9–12 months) over the 12-month period. Estimates are derived from linear mixed-effects models with participant-level random intercepts; each participant’s repeated measurements therefore contribute to the modeled trajectory.

A consistent and statistically significant weight reduction and maintenance was demonstrated across all time intervals (0–3, 3–6, 6–9, and 9–12 months; *p* < 0.001 for all comparisons). During the initial 0-to-3-month period, participants experienced an average monthly weight loss of 0.88 kg (95% CI: −0.956 to −0.812, *p* < 0.001), corresponding to a cumulative reduction of 2.65 kg. This effect intensified during months 3 to 6, with an estimated monthly loss of 1.13 kg (95% CI: −1.21 to −1.047, *p* < 0.001), yielding a total reduction of 3.40 kg for this interval. From 6 to 9 months, the rate of weight loss slowed to 0.80 kg per month (95% CI: −0.917 to −0.688, *p* < 0.001), corresponding to a further reduction of 2.40 kg. Finally, in the 9-to-12-month period, weight loss continued at a diminished pace of 0.56 kg per month (95% CI: −0.659 to −0.453, *p* < 0.001), resulting in an additional average reduction of 1.67 kg (Figure 2). When aggregating these effects, the estimated cumulative weight loss over the entire 12-month period was 10.12 kg (11.4% of baseline body weight). BMI reduction followed the same pattern, with a cumulative decrease of 3.68 kg/m^2^ by the end of the 12-month program.

Age showed a negative association with weight and BMI reduction, although significance was inconsistent across models. Sex was not significantly associated with weight loss in any period. No interaction effects by age or sex on time-dependent weight loss were observed.

### 3.3. Blood Biomarker Changes Following Precision Nutrition

**Figure 3:**
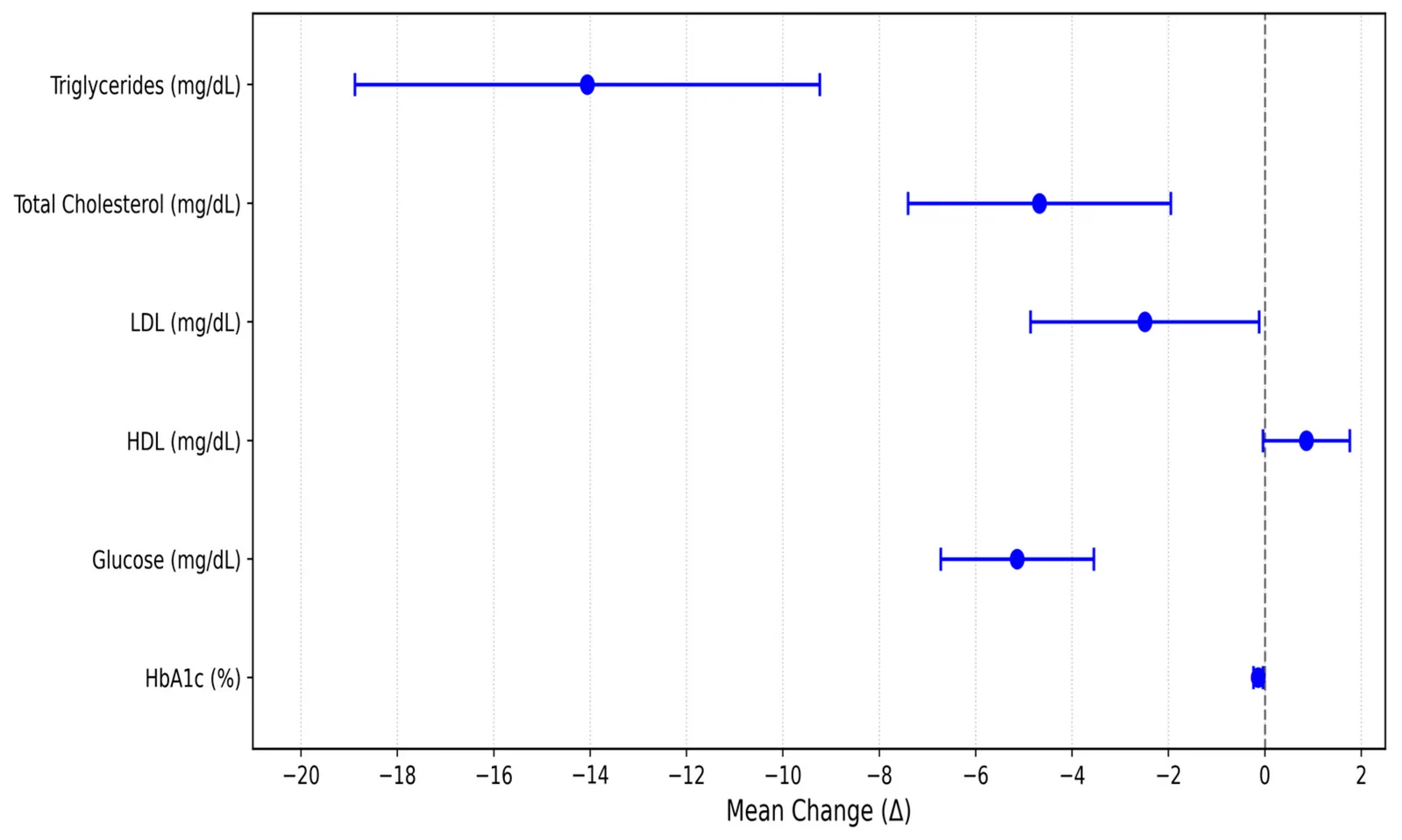
Mean changes in blood biochemical biomarkers following a 12-month nutrigenetic diet. Mean changes (Δ) from baseline to follow-up are shown (blue circles) for Triglycerides (mg/dL), Total Cholesterol (mg/dL), LDL Cholesterol (mg/dL), HDL Cholesterol (mg/dL), Glucose (mg/dL), and HbA1c (%). Horizontal bars indicate 95% confidence intervals. The vertical dashed line at zero represents no change. Negative values indicate reductions from baseline.

**Figure 4:**
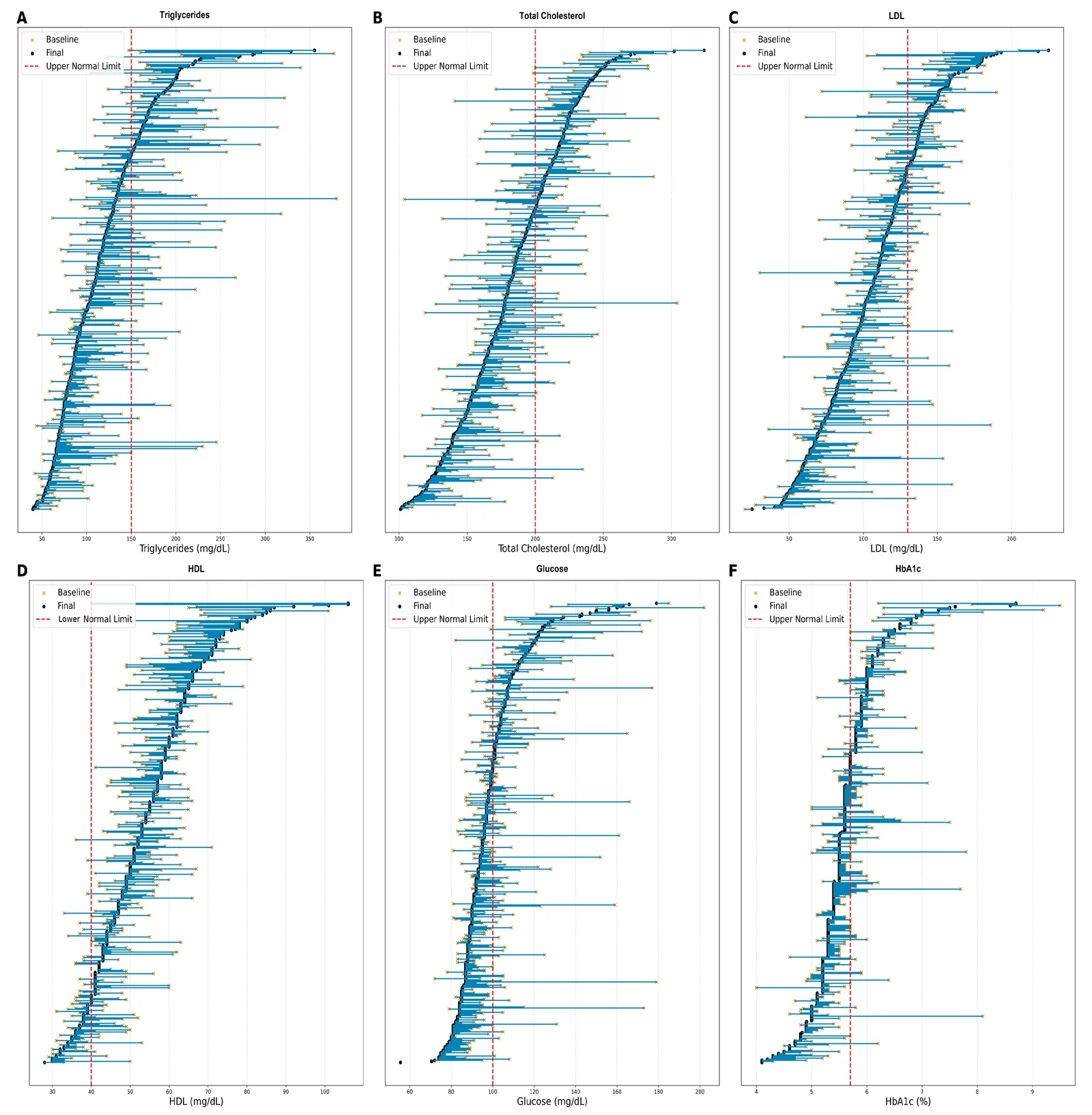
Paired lollipop plots of biomarker changes. Baseline (orange ‘x’) and Final (black circles) individual values are presented for (**A**) Triglycerides, (**B**) Total Cholesterol, (**C**) LDL Cholesterol, (**D**) HDL Cholesterol, (**E**) Glucose, and (**F**) HbA1c. Blue lines connect paired values for individual participants. Red dashed lines indicate the clinical upper normal limit for each biomarker, except for HDL cholesterol, where the lower normal limit is shown, as higher HDL values are favorable.

Among 426 participants who completed both baseline and follow-up blood testing over the course of 12 months, a simultaneous analysis of all biomarkers revealed a significant main effect of Time (F = 10.30, *p* < 0.001, Wilks’ Λ = 0.87, partial η^2^ = 0.129). No significant interaction was observed between Time and Sex, indicating that males and females exhibited similar patterns of change over the follow-up period. Subsequent univariate analyses confirmed statistically significant and clinically relevant improvements in five biomarkers: TG, total cholesterol, LDL cholesterol, glucose, and HbA1c (Figure 3 and Figure 4).

Changes were observed across multiple lipid parameters. Of all the biomarkers assessed, triglycerides showed the greatest improvement. Across the full cohort, TG levels decreased by an average of 14.06 mg/dL (SE = 2.46), a statistically significant reduction (F = 32.7, *p* < 0.001, partial η^2^ = 0.072). At baseline, 140 participants had elevated TG levels (≥150 mg/dL). Following the program, 65 of these individuals (46.4%) transitioned into the normal range, indicating a clinically meaningful improvement in TG regulation.

Total cholesterol levels showed a significant decrease, with an average reduction of 4.68 mg/dL (SE = 1.39), a statistically significant change (F = 11.30, *p* = 0.001, partial η^2^ = 0.026). At baseline, 160 participants had elevated total cholesterol (≥200 mg/dL), and after 12 months, 47 of them (29.4%) improved into the normal range. LDL cholesterol levels also declined, with an average reduction of 2.49 mg/dL (SE = 1.21), representing a modest but statistically significant reduction (F = 4.27, *p* = 0.039, partial η^2^ = 0.010). At baseline, 119 participants had elevated LDL cholesterol (≥130 mg/dL), and by the 12-month follow-up, 41 individuals (34.5%) transitioned into the normal range.

A small overall increase was observed in HDL cholesterol across the cohort. On average, HDL increased by 0.86 mg/dL (SE = 0.46), a change that did not reach statistical significance (F = 3.51, *p* = 0.062, partial η^2^ = 0.008). Despite this slight numerical rise, HDL levels remained relatively stable over the follow-up period, with no statistically significant improvement at the population level.

Markers of glycemic control also improved. Across the entire cohort, fasting glucose levels declined by an average of 5.14 mg/dL (SE = 0.81), a statistically significant reduction (F = 39.76, *p* < 0.001, partial η^2^ = 0.086). At baseline, 207 participants had elevated glucose levels (≥100 mg/dL). By the 12-month follow-up, 82 individuals (39.6%) achieved values in the normal range. Similarly, HbA1c levels decreased modestly but significantly, with an average reduction of 0.14% (SE = 0.05; F = 9.33, *p* = 0.002, partial η^2^ = 0.022). At baseline, 234 participants had elevated HbA1c levels (≥5.7%), and by 12 months, 83 participants (35.5%) transitioned into the normal range. The proportion of participants with abnormal baseline values who normalized by 12 months is summarized in Table 2.

## 4. Discussion

The findings of this real-world, community-based retrospective data study indicate that a nutrigenetic-based dietary program, integrated with established nutritional principles and supported by initial structured counseling, is associated with sustained weight loss and meaningful improvements in metabolic risk indicators over a 12-month follow-up. Participants achieved an average weight reduction of 10.12 kg (11.4% of baseline) at 12 months, alongside a progressive decline in BMI (total decrease of 3.68 kg/m^2^) with a continuous reduction rate. The program was also linked to favorable changes in cardiometabolic biomarkers; specifically, significant improvements in TG, total cholesterol, LDL cholesterol, fasting glucose, and HbA1c, with a substantial proportion of participants normalizing previously elevated values.

Weight reduction was significant and consistent throughout the follow-up period, with no plateau observed within the first year. Weight loss and maintenance differed between periods, with the greatest monthly reduction during months 3–6 and a gradual attenuation of the rate thereafter. This sustained progress suggests that compliance with the nutrigenetic personalized weight management programs was significant, achieving long-term lifestyle changes and resulting in sustained weight loss and improved blood biochemical levels. Furthermore, the structured early-phase counseling by clinical dietitians translated genetic insights into practical strategies, while continuous self-monitoring fostered accountability and reinforced habit formation over time.

When considered alongside published personalized-nutrition intervention studies (including both observational studies and RCTs), the magnitude of weight loss in our cohort is comparable to or falls within the upper range of previously reported outcomes. Notably, the results of our study show sustained weight reduction over 12 months in a real-world community setting, reflecting a challenging, uncontrolled obesogenic environment, which could have hindered weight reduction and maintenance. This contrasted with prior interventions, in which weight regain often commenced within the first year; in our cohort, weight loss in a substantial proportion of participants continued progressively throughout the 12-month follow-up, rather than regressing toward baseline weight.

Several clinic-based, non-randomized intervention studies have reported short-term weight loss following the incorporation of nutrigenetic dietary guidance, with results comparable to those observed in our cohort. For example, Lakshmi et al. (*n* = 69) reported a mean weight reduction of 5.0 kg over 4 months among participants following a nutrigenetic diet, whereas no significant change was observed in the generic diet or control arms [44]. Similarly, Tamilvanan et al. (*n* = 106) reported a mean reduction of 4.8 kg in the nutrigenetic group compared with 2.3 kg in controls after 120 days, alongside higher adherence in the personalized intervention arm [45].

Results from the few randomized controlled trials (RCTs) conducted to date have been mixed. Frankwich et al. (*n* = 51) reported similar mean reductions of approximately 5.0 kg in both nutrigenetic and control groups after 24 weeks [39]. In the POINTS trial (*n* = 145), no statistically significant difference was found between participants assigned to genotype-concordant diets versus conventional diets after 12 weeks (5.3 kg vs. 4.8 kg, respectively) [43]. The PREVENTOMICS trial (*n* = 100) also found no difference between groups, with both the omics-based plan and control diet yielding comparable weight reductions of approximately 3 kg over 10 weeks [42]. Furthermore, in the NOW trial (*n* = 140), while both groups achieved early weight loss, participants subsequently regained weight, resulting in modest net reductions by 12 months (−2.94 kg in the nutrigenomics group vs. −2.86 kg in standard care) [40]. This contrasts with the sustained weight loss observed in our cohort.

The large-scale Food4Me trial (*n* = 1607) also reported no added benefit of genetic personalization on weight loss over standard dietary advice after 6 months (−0.5 kg to −0.7 kg across arms) [54]. However, it is important to note that Food4Me enrolled a generally healthy adult cohort (mean BMI 25.5 kg/m^2^) and was designed primarily to assess changes in dietary behavior using an automated online feedback framework. Consequently, the modest weight changes observed are consistent with the study’s specific aims and population structure, which differ significantly from the clinically obese cohort in our study.

Beyond nutrigenetics, conventional lifestyle and dietary interventions in primary care and community settings typically yield more modest outcomes compared to our findings. Meta-analyses of randomized controlled trials consistently report a mean weight loss of 2–3 kg at 12 months [23,55,56]. Furthermore, clinical guidelines establish that a 5–10% reduction in body weight at 12 months is generally considered clinically meaningful [20]. In this context, the greater reductions observed in our cohort may reflect the potential added value of combining personally adjusted nutrigenetic information in dietary recommendation beyond the standard recommendations and underscore the need to further investigate the mechanisms underlying this sustained effect.

The significant improvements in metabolic indicators observed in this study are consistent with the physiological expectations following substantial weight loss, as documented by The Look AHEAD Research Group [57]. However, beyond the effect of weight reduction alone, these favorable outcomes may be partly related to the precision of the nutrigenetic approach. Beyond caloric restriction, genotype-specific recommendations are designed to optimize nutrient–gene interactions [58,59], which may further enhance metabolic stabilization and improve long-term adherence. To our knowledge, longitudinal cardiometabolic data from such personalized interventions over a full 12-month period remain scarce, limiting direct comparisons. Consequently, we contextualize our findings against conventional dietary trials that evaluated similar biomarkers, while acknowledging the inherent differences in study populations.

TG levels demonstrated the most substantial improvement among all measured biomarkers, decreasing by a mean of 14.06 mg/dL at 12 months. Notably, 46.4% of participants with elevated baseline values transitioned into the normal range. These substantial reductions are consistent with data from key randomized controlled trials (RCTs), though the methodological settings and participant profiles differ. For instance, the DIETFITS trial (*N* = 609) evaluated a significantly younger cohort (mean age 40 ± 7 years; mean BMI 33 ± 3 kg/m^2^). Its protocol was exceptionally intensive, involving 22 group instructional sessions (60–90 min each), and reported TG reductions of 9.95 mg/dL in the low-fat arm and 28.20 mg/dL in the low-carbohydrate arm over 12 months [26]. Similarly, the PREDIMED-Plus trial (*N* = 626 subgroup), which assessed older adults (aged 55–75) with metabolic syndrome and a mean BMI of 32.5 ± 3.4 kg/m^2^, reported a TG reduction of 15.1 mg/dL. This intervention followed a rigorous protocol of an energy-restricted Mediterranean-style diet combined with physical activity promotion and intensive behavioral support consisting of three monthly contacts [60]. In contrast, our cohort represents a real-world demographic with a higher mean age (63.21 ± 11.08 years) and a comparable high baseline BMI (32.11 ± 4.58 kg/m^2^). Taken together, while these RCTs relied on high-frequency clinical contact and strict dietary restrictions, our findings suggest that comparable metabolic improvements can be achieved in a community setting through a flexible, personally adjusted nutrigenetic protocol. By allowing for personalized food choices and requiring less clinical manpower, our approach offers a scalable and sustainable model for long-term metabolic management.

In our study, fasting glucose levels declined by a mean of 5.14 mg/dL at 12 months, with 39.6% of participants who presented with elevated baseline values (≥100 mg/dL) returning to the normal range. Similarly, HbA1c levels decreased by 0.14% at 12 months, and 35.5% of those with elevated baseline values (≥5.7%) achieved levels within the reference range. This finding aligns with systematic evidence; a recent meta-analysis of 7 RCTs involving 1371 patients with type 2 diabetes, which assessed Mediterranean-style interventions over at least 6 months, demonstrated a pooled fasting glucose reduction of 15.12 mg/dL and HbA1c reduction of 0.39% [61]. While the magnitude of reduction in our non-diabetic cohort was expectedly lower than in these diabetic populations, the direction of improvement is consistent.

Our study results compare favorably to specific large-scale trials. Specifically, fasting glucose reduction (5.14 mg/dL) exceeded the decreases reported in the DIETFITS trial (*N* = 609), which were 3.67 mg/dL in the low-fat arm and 2.10 mg/dL in the low-carbohydrate arm [26]. Similarly, our findings mirror the PREDIMED-Plus trial (*N* = 626), which reported reductions of 4.1 mg/dL in glucose and 0.12% in HbA1c following 12 months of an energy-restricted Mediterranean diet [60]. These glycemic improvements in an older real-world demographic are challenging to compare with the 22 instructional sessions of DIETFITS or the strict energy restrictions of PREDIMED-Plus, and are indicative of the efficiency of our nutrigenetic approach.

After 12 months, total cholesterol levels decreased by a mean of 4.68 mg/dL, and notably, 29.4% of participants with elevated baseline values (≥200 mg/dL) returned to the reference range by 12 months. This reduction aligns with findings from the PREDIMED-Plus trial, where participants in the intervention group experienced a comparable mean reduction of approximately 5.0 mg/dL after 12 months [60]. LDL cholesterol levels decreased by a mean of 2.49 mg/dL, with 34.5% of participants with elevated baseline values (≥130 mg/dL) achieving values within the reference range after 12 months. Broader evidence from a network meta-analysis of 121 RCTs (*n* = 21,942) evaluating 14 popular named dietary programs (including Atkins, DASH, and Mediterranean) suggests that maintaining LDL reductions is challenging. While modest reductions were observed at approximately 6 months (e.g., low-fat: 7.08 mg/dL; moderate-macronutrient: 5.22 mg/dL; Mediterranean: 4.59 mg/dL), by 12 months, improvements in cardiovascular risk factors largely diminished for most dietary patterns, with the exception of the Mediterranean diet, which retained some benefit [62]. Specific trial data supports this; our reduction mirrors findings from the DIETFITS trial, where LDL decreased by 2.12 mg/dL in the low-fat arm, while increasing by 3.62 mg/dL in the low-carbohydrate arm [26].

HDL cholesterol levels exhibited a small mean increase of 0.86 mg/dL at 12 months, which did not reach statistical significance. Broader evidence from the same network meta-analysis is consistent with the difficulty of achieving sustained HDL improvements in such settings, demonstrating that by 12 months, no dietary pattern maintained a significant increase in HDL levels compared to a usual diet [62]. This aligns with findings from large-scale free-living trials; for instance, the DIETFITS trial reported similarly modest HDL increases ranging from 0.40 to 2.64 mg/dL, depending on the diet arm [26].

This study presents several notable strengths. First, the relatively large cohort sample size for the nutrigenetic program, combined with the extended 12-month follow-up and the community-based setting, provides real-world evidence. Second, the integration of genetic, medical, biochemical, and clinical data enabled a comprehensive evaluation of how precision nutrition can be implemented in practice, yielding results comparable in magnitude to those reported in controlled clinical trials. Third, outcomes were not limited to weight reduction but encompassed multiple biochemical markers, providing a multidimensional assessment of metabolic health. Methodologically, the application of linear mixed-effects modeling allowed for the adjustment of repeated measures and covariates (age, sex), enabling the capture of weight change trajectories across specific intervals rather than relying solely on pre–post comparisons. Finally, the analysis of normalization rates, defined as the proportion of participants with abnormal baseline values who achieved values within clinically recommended ranges, provides a clinically meaningful perspective that has not been systematically reported in prior nutrigenetic studies. Importantly, the longitudinal mixed-effects framework meant that each participant effectively served as their own control, with change modeled relative to their individual baseline. This within-subject design accounts for stable inter-individual differences, including genetic, demographic, and lifestyle factors, that frequently confound between-group comparisons, thereby partially mitigating the absence of a parallel control arm, although it does not permit causal inference.

Several limitations should be acknowledged. As an observational, real-world study, the design precludes definitive conclusions regarding causality, and the absence of a randomized control group limits the ability to directly compare outcomes against non-personalized dietary interventions. Furthermore, weight data were primarily self-reported; although periodic verification by dietitians mitigated this potential bias, it could not be eliminated. The cohort was also predominantly female and older, which may restrict the generalizability of the findings to younger populations or men. Finally, selection bias is a consideration, as participants who voluntarily purchased the program and consistently engaged with the application likely represent a more motivated subset of the population, potentially differing from the general public in their readiness for behavior change. Notably, the final analytic cohort represented approximately 4% of the initial database. This marked reduction was primarily a direct consequence of the strict, multi-step inclusion criteria applied, chiefly the requirement for at least two valid, dietitian-verified weight measurements and two complete blood biochemical records at both baseline and 12-month follow-up, rather than selective dropout from the program itself; the majority of excluded individuals were removed at this data-completeness stage. This conservative approach prioritized the reliability and completeness of the longitudinal data over sample size, thereby strengthening the internal validity of the observed outcomes, although it may limit generalizability to the broader consumer population.

Although a qualitative dietary anamnesis was obtained at intake and dietary habits were discussed during follow-up, no structured, validated quantitative assessment of dietary intake was performed. Consequently, adherence to the prescribed plan and actual energy and macronutrient intake could not be quantified. Furthermore, although lifestyle factors such as physical activity and sleep were addressed by the dietitian during counseling as part of routine care, they were not systematically measured or recorded as study variables, and were therefore not included as covariates in the analysis. Their potential confounding effect on the observed outcomes therefore cannot be excluded. Notably, because the program was self-selected and combined genetic personalization with paid dietitian support and participant motivation, the present design cannot isolate the specific contribution of the nutrigenetic component from these accompanying factors; it therefore remains possible that comparable outcomes could be achieved through structured dietitian-led counseling without a genetic basis, and a randomized comparison against equivalent non-genetic support is needed to establish the added value of the nutrigenetic approach. Waist circumference was not recorded in this retrospective dataset; consequently, the waist-to-height ratio could not be computed. To assess potential selection bias, the final cohort was compared with participants who did not meet the data-completeness criteria; the groups were similar in sex (66.9% vs. 66.7% female) and baseline weight (89.0 vs. 87.6 kg), with a modestly higher baseline BMI in the final cohort (32.1 vs. 31.4 kg/m^2^). A sensitivity analysis in a broader group (initial BMI > 25; *n* = 1525, and *n* = 689 with at least two blood measurements) yielded a consistent pattern of weight loss, indicating that the findings are robust to the inclusion criteria.

## 5. Conclusions

From a practical perspective, our findings suggest that personalized dietary interventions anchored in genetic information may provide a scalable approach to achieving sustained weight loss and improvements in metabolic markers. Such programs could complement existing lifestyle modification frameworks in both clinical and community settings by offering targeted counseling alongside behavioral support, while allowing flexible, nutrigenetic food choices rather than rigid dietary prescriptions or intensive follow-up. Future research should examine whether specific genetic- or biomarker-defined subgroups derive greater benefit; understanding which profiles are most responsive is essential to prioritize interventions, optimize cost-effectiveness, and guide implementation at scale.

In summary, a nutrigenetic-based dietary program implemented in a community setting, followed by self-directed monitoring, was associated with sustained, clinically meaningful weight loss and metabolic improvements at 12 months. The ability to achieve these outcomes without strict dietary regimens or continuous clinical supervision supports the practicality of this flexible model. Together, these results provide preliminary real-world evidence that integrating genetic information into nutrition counseling may be a feasible and promising approach for improving health and adherence to diet. More research, including controlled studies, is warranted to further advance the application of personalized nutrition.

## Figures and Tables

**Table 1:** Cohort baseline characteristics.

Variable (Mean ± SD)	Cohort (*n* = 426)
Sex (% females)	285 (66.9%)
Age (years)	63.21 ± 11.08
Weight (kg)	88.98 ± 15.90
Height (cm)	166.23 ± 8.94
BMI (kg/m^2^)	32.11 ± 4.58
Triglycerides (mg/dL)	132.06 ± 61.06
Total Cholesterol (mg/dL)	187.44 ± 40.09
LDL Cholesterol (mg/dL)	109.07 ± 32.80
HDL Cholesterol (mg/dL)	52.75 ± 11.54
Glucose (mg/dL)	104.34 ± 24.12
HbA1c (%)	5.81 ± 0.95

**Table 2:** Normalization of cardiometabolic biomarkers following the 12-month nutrigenetic program. Values are the number and percentage of participants with an abnormal baseline value, and, among them, the number and percentage who reached the clinical reference range at 12 months. HDL cholesterol is not shown, as its change was not statistically significant. Percentages in the last column are calculated within the baseline-abnormal subgroup.

Biomarker	Abnormal Cut-Off	*n* (%) Abnormal at Baseline	*n* (%) Normalized at 12 Months
Triglycerides	≥150 mg/dL	140 (32.9%)	65 (46.4%)
Total cholesterol	≥200 mg/dL	160 (37.6%)	47 (29.4%)
LDL cholesterol	≥130 mg/dL	119 (27.9%)	41 (34.5%)
Fasting glucose	≥100 mg/dL	207 (48.6%)	82 (39.6%)
HbA1c	≥5.7%	234 (54.9%)	83 (35.5%)

## Data Availability

The data are not publicly available due to privacy reasons.

## Supplementary Materials

The following supporting information can be downloaded at: https://www.mdpi.com/article/10.3390/nu18162618/s1, Figure S1: The Mediterranean-style dietary pattern on which the program was based, illustrated as a food pyramid by recommended frequency of intake; Figure S2: Study design and schedule of assessments; Table S1: Specific food recommendations within the Mediterranean-style dietary framework applied in the program.

## Abbreviations

The following abbreviations are used in this manuscript: Analysis of variance (ANOVA); Body mass index (BMI); Confidence interval (CI); Deoxyribonucleic acid (DNA); Direct-to-consumer (DTC); Fat mass and obesity-associated gene (*FTO*); Genome-wide association studies (GWAS); Glycated hemoglobin (HbA1c); High-density lipoprotein (HDL); Health maintenance organization (HMO); Low-density lipoprotein (LDL); Multivariate analysis of variance (MANOVA); Peroxisome proliferator-activated receptor gamma (*PPARG*); Randomized controlled trial (RCT); Restricted maximum likelihood (REML); Standard deviation (SD); Standard error (SE); Single-nucleotide polymorphism (SNP); Triglycerides (TG); World Health Organization (WHO).
