# Supplementary material for: Nutrigenetic Tailored Diet Results in Long-Term (12 Months) Weight-Loss Compliance and Improved Cardiometabolic Outcomes in a Real-World Community-Based Setting
Source: Nutrients. 2026 Aug 10;18(16):2618. doi: 10.3390/nu18162618 (PMC13516270; doi:10.3390/nu18162618)
Supplement: Supplementary file 1 [file nutrients-18-02618-s001.zip › nutrients-4415473-supplementary.pdf]

Supplementary Materials

The personalized dietary guidance in this study was based on a Mediterranean-style dietary pattern, as defined by national (Israeli Ministry of Health) and international consensus recommendations [47,48,52], and was individually tailored to each participant's nutrigenetic test recommendations. Figure S1 presents this framework as a food pyramid, organized by the recommended frequency of intake, and Table S1 details the specific food recommendations within it.

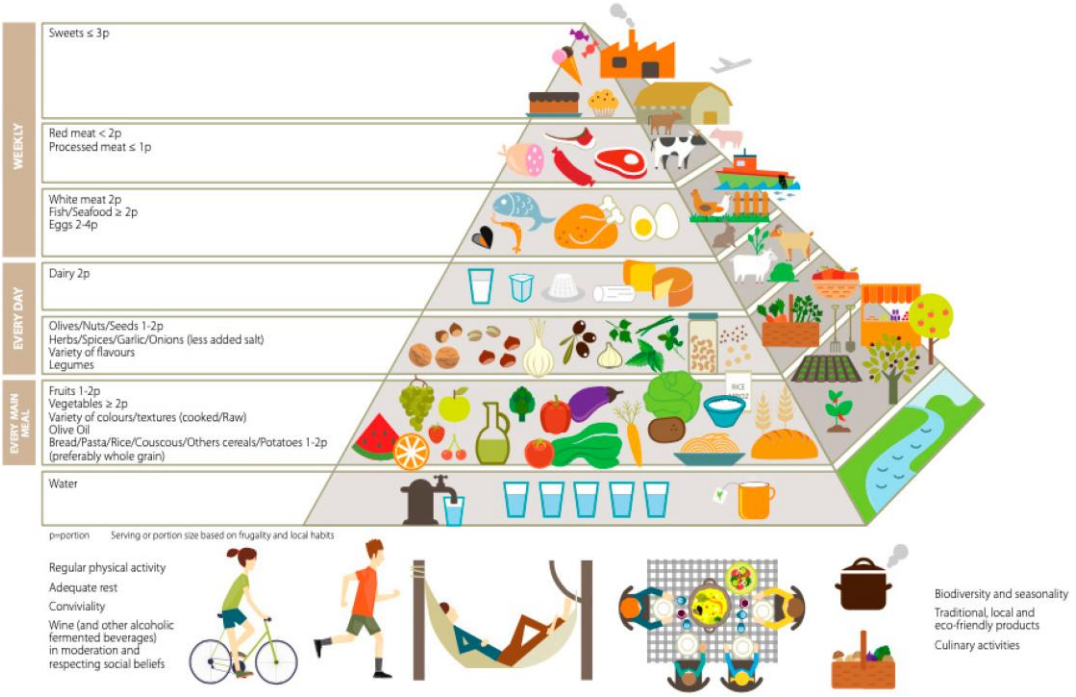

Figure S1. The Mediterranean-style dietary pattern on which the program was based, illustrated as a food pyramid by recommended frequency of intake. Within this framework, each participant's food choices were individually tailored to their nutrigenetic test recommendations. Reproduced from Serra-Majem et al. [53], under a Creative Commons Attribution (CC BY 4.0) license.

Table S1. Specific food recommendations within the Mediterranean-style dietary framework applied in the program.

Food group	Recommended choices and amounts
Vegetables and fruits	≥5 servings/day of varied, colorful, unpeeled produce; included in most main meals, with seasonal produce preferred. Fruit encouraged daily as the default dessert.
Whole grains	Whole-wheat bread, oats, and quinoa; preferred over refined grains as the main carbohydrate source.
Legumes	Lentils, chickpeas, and beans, encouraged as a regular plant-protein source.
Healthy fats	Olive oil as the main source of dietary fat; also avocado, almonds, tahini, and canola oil. Added oils used sparingly.

Fish and seafood	Omega-3–rich fish (e.g., salmon, tuna, sole), emphasized as a regular protein source.
Poultry and eggs	Lean poultry in moderation; eggs in moderation.
Dairy	Unsweetened, low-fat dairy (mainly yogurt and cheese) or sugar-free soy alternatives.
Nuts and seeds	Moderate, unsalted portions.
Red and processed meat	Limited; lean beef ≤300 g/week. Avoid high-fat and processed meats.
Added sugars	Avoid foods listing sugar, honey, or syrups high on the ingredient panel.
Ultra-processed foods	Avoid industrial baked goods and foods with synthetic additives.
Salt and sodium	<2400 mg/day; reduced salt, with herbs and spices used for flavoring. Limit salty snacks.
Beverages	Water as the main beverage; avoid sugary drinks, fruit juices, flavored waters, and energy drinks.
Alcohol	Minimize consumption.

Within this framework, individual food choices were tailored to each participant's nutrigenetic test recommendations, emphasizing flexible, genetically informed choices rather than a single fixed dietary prescription.

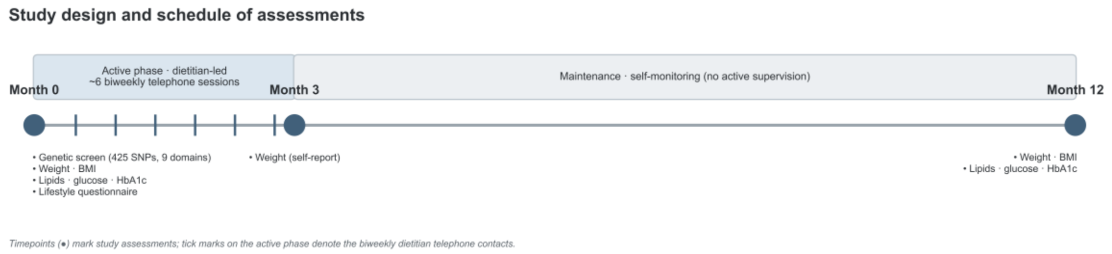

Figure S2. Study design and schedule of assessments. Participants underwent direct-to-consumer genetic screening (425 SNPs across several domains) and a baseline assessment by a registered dietitian, followed by a 3-month active phase with approximately six biweekly telephone sessions and a subsequent 9-month self-monitoring maintenance phase. Anthropometric measures (weight, BMI) and blood biochemical markers (triglycerides, total cholesterol, LDL cholesterol, HDL cholesterol, glucose, HbA1c) were obtained at baseline and at 12 months. Tick marks on the active phase denote the biweekly dietitian telephone contacts.
